# Prevalence of frailty in a tertiary hospital: A point prevalence observational study

**DOI:** 10.1371/journal.pone.0219083

**Published:** 2019-07-01

**Authors:** Simon J. G. Richards, Joel D’Souza, Rebecca Pascoe, Michelle Falloon, Frank A. Frizelle

**Affiliations:** Department of Surgery, University of Otago, Christchurch, New Zealand; University of Notre Dame Australia, AUSTRALIA

## Abstract

**Introduction:**

Frailty is an important concept in modern healthcare due to its association with adverse outcomes. Its prevalence varies in the literature and there is a paucity of literature looking at the prevalence of frailty in an inpatient setting. Its significance lies on its impact on resource utilisation and costs.

**Aim:**

To determine the prevalence of frailty in the adult population in a tertiary New Zealand hospital.

**Methods:**

Eligible patients aged 18 years and over were invited to participate, and frailty assessment was performed using the Reported Edmonton Frail Scale. A score of 8 or more was considered frail. Factors associated with frailty were assessed.

**Results:**

Of 640 occupied inpatient beds, 420 patients were assessed. 220 patients were excluded, of which 89 were absent from their bed-space, 73 declined and 41 were critically unwell. The overall prevalence of frailty across assessed patients was 48.8%. The prevalence of frailty increased significantly with age; patients aged 85 and over were significantly more likely to be frail compared to those aged under 65 (OR 6.25, 95% CI 3.17–12.7). Maori patients were significantly more likely to be frail (OR 4.0, 95% CI 1.45–11.9). When compared to those patients admitted to a medical specialty, patients admitted to surgical specialty were less likely to be frail (OR 0.52 95% CI 0.31–0.86) and those admitted for rehabilitation were more likely to be frail (OR 1.86 95% CI 1.03–3.41). Frail patients were more likely to come from a rest home (OR 2.81, 95% CI 1.38–6.14) or hospital level care (OR 9.62, 95% CI 2.68–61.6).

**Conclusion:**

Frailty is highly prevalent in the hospital setting with 48.8% of all inpatients classified as frail. This high number of frail patients has significant resource implications and an increased understanding of the burden of frailty in this population may aid targeting of interventions towards this vulnerable population.

## Introduction

Frailty is an important concept in modern healthcare due to its association with adverse health outcomes, prolonged hospital admissions and discharge to residential care facilities[1–6]. Frailty can be conceptualised as a state of increased vulnerability resulting from age-associated declines in physiological reserves and function across multiple organ systems, such that the ability to cope with every-day or acute stressors is compromised[7–9].

The prevalence of frailty in the literature varies considerably, typically as a result of varying populations, definitions and diagnostic criteria. Despite the need to identify and measure frailty, there is no single operational definition for clinical use[10], therefore making interpretation and comparison of literature difficult. Collard et al[11], in a systematic review of 21 community based cohort studies, reported rates varying between 4% and 59.1%, with an overall weighted prevalence of 10.7% in an elderly population. The Survey of Health, Ageing and Retirement in Europe (SHARE) study reported a frailty prevalence of 4.1% in community dwelling individuals aged over 50 and of 17% in those aged over 65 across 10 European countries[12].

The prevalence of frailty in hospitalised elderly patients also varies significantly in the literature with reported rates between 24.7% and 80%[13–18]. In a cohort of 220 patients aged 70 years and older who were admitted to an acute geriatric ward Joosten et al[13] identified 40% of individuals as frail and 51.5% as pre-frail. In 307 patients admitted with a myocardial infarction and aged 75 years and older 48.8% were identified as frail. Typically, frailty prevalence assessment focusses on single service or demographic and provided useful information on that specific cohort. To our knowledge no prospective study has been performed assessing the prevalence of frailty across an entire hospital.

Multiple frailty assessment tools exist for use in both research and clinical settings.[19] The Edmonton Frail Scale (EFS) is a multidimensional assessment tool that includes assessment across different domains[20]. It takes less than five minutes to perform and is feasible for use by non-geriatricians[21] It is scored out of seventeen and has nine components including general heath, self-reported health, cognition, functional independence, social support, mood, polypharmacy, continence and functional performance. It is valid, reliable and its diagnostic accuracy has been reviewed in the hospital setting[20, 22, 23]. Due to the potential difficulty of performing a timed up and go test on hospital inpatients, an adaptation of the EFS, the reported Edmonton Frail Scale (REFS) was created by Hilmer et al[24]. Instead of a timed up and go test, the REFS utilises a self-reported performance state, two weeks prior to admission. Hilmer et al were able to demonstrate the reliability and validity of the REFS in acute patients admitted to a Australian hospital[24].

The aim of this study was to evaluate the prevalence of frailty in adult inpatients in a tertiary hospital. Although there are other well validated frailty indexes, the REFS was chosen due to its multidimensional nature, simplicity and its validity in the hospital setting.

## Methods

### Patient population

Christchurch Hospital is the largest tertiary hospital in the South Island of New Zealand with 580 adult inpatient beds with a further 230 beds at the nearby Burwood Health Campus. Burwood Health Campus is predominantly a rehabilitation facility, however, does have some elective surgical beds. Typical district health board bed occupancy is between 70 and 80%. On a typical day, based on inpatient prediction models, all inpatients aged 18 and over at Christchurch and Burwood Hospitals were invited to participate in a point prevalence assessment of frailty. Patients in the emergency department, birthing suite and intensive care units were excluded from the study due to the acuity of their conditions.

Patients were identified for inclusion in the study by inpatient lists generated by the Decision Support team at 0730am on the day of assessment. This list was cross referenced by the assessor at the time of assessment to ensure patients who moved location during the day were not duplicated.

Demographic data including age, gender, ethnicity, admission service and mode of admission (acute vs elective) was provided by Decision Support from the data warehouse. Data on level of care was obtained by the assessor at the point of recruitment.

### Frailty assessment

Frailty assessment was performed using the Reported Edmonton Frail Scale[24] by 24 trained medical and nursing staff who were assigned in pairs to different wards. Frailty assessment questions were referenced to the time of admission, in an attempt to standardise given varying admission lengths at time of assessment. For example, medication usage was asked with regard to the time of admission, rather than at the time of assessment.

On initial contact a patient information sheet and a verbal description of the study was provided. Written consent was obtained, baseline demographics collected, and frailty assessment completed. Results of all individual components of the REFS were collected and a score between 0 and 18 was obtained. Any individual having a score of 8 or greater on the Reported Edmonton Frail Scale will be deemed as being frail. Patients were classified as having mild frailty with a score of 8 to 9, moderate frailty with a score of 10 to 11 and severe frailty with a score of 12 to 18[24]. Patients scoring 6 to 7 on the Reported Edmonton Frail Scale were classified as apparently vulnerable.

Due to a presumed high prevalence of frailty in confused patients it was felt to be important to include this cohort in our analysis. Confused patients were those who had either an acute confusional state, such as delirium, or a chronic confusional state, such as dementia, and were alerted to assessors by either the medical staff, or by the inability of the assessor to obtain consent. If a patient was confused or unable to provide consent the assessor alerted one of the primary investigators who subsequently contacted the lead clinician to obtain clarification of the diagnosis of confusion and to obtain consent to complete the assessment by review of clinical notes and discussion with the patients next of kin.

### Ethics

This study was approved by the New Zealand Health and Disability Ethics Committee reference 18/STH/173. Locality approval was obtained from the Canterbury District Health Board.

### Statistical analysis

Continuous variables were reported as medians with interquartile range (IQR) and categorical variables as whole numbers and percentages. Univariable analysis was performed to compare baseline characteristics between frail and non-frail patients. This included chi-square tests for categorical variables and Kruskal-Wallis test for non-parametric continuous variables. The distribution of continues variables were tested for parametricity using data visualization and the Shapiro-Wilk normality test. Multicollinearity between predictor variables was assessed by variance inflation factor (VIF), with scores of over 3 taken to denote unacceptable collinearity.

Multivariable logistic regression analysis was performed to identify sociodemographic factors predictive of frailty using backward stepwise selection based on Akaike Information Criterion (AIC). Model performance was assessed using Harrell’s concordance index (C-index). Regression coefficients from multivariable analysis were reported as odd’s ratios with 95% confidence intervals (95% CI). P values <0.05 were considered statistically significant. All statistical analysis was performed using RStudio software (version 3.3.2; www.rstudio.com).

## Results

On the day of assessment, 640 patients, representing 79% of the total hospital occupancy were eligible for inclusion. This included 475 patients from the Christchurch Hospital Campus and 165 from the Burwood Hospital Campus. Of the 640 eligible patients, 386 provided consent and a further 34 confused patients were subsequently included with the consent of their lead clinician. This resulted in a total of 420 patients (65.6%) being included in this study.

**Fig 1:**
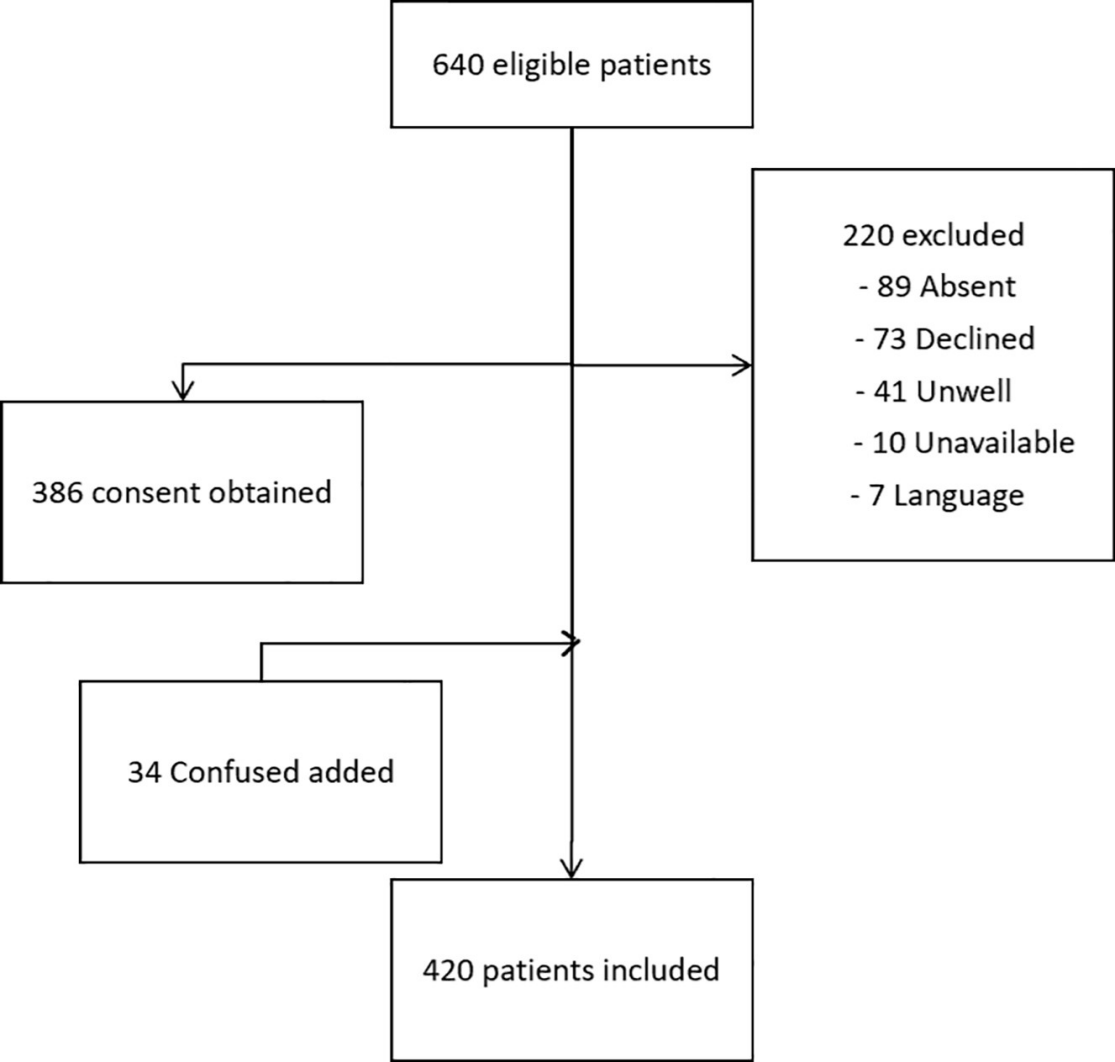
Patient recruitment overview.

Reasons for the exclusion of the remaining 220 patients included 89 patients (13.9%) being absent from their bed-space, 73 (11.4%) patients declining, 41 (6.4%) patients who were either critically unwell or receiving end of life cares, 10 (1.6%) patients who were with other health care professionals and 7 (0.9%) patients who declined due to language difficulties. Of the 89 patients absent from their bed-space, 22 were in an operating theatre, 17 were at investigations and the remaining 50 were presumed to be ambulatory and off the ward. The final cohort included 420 patients (Fig 1).

**Fig 2:**
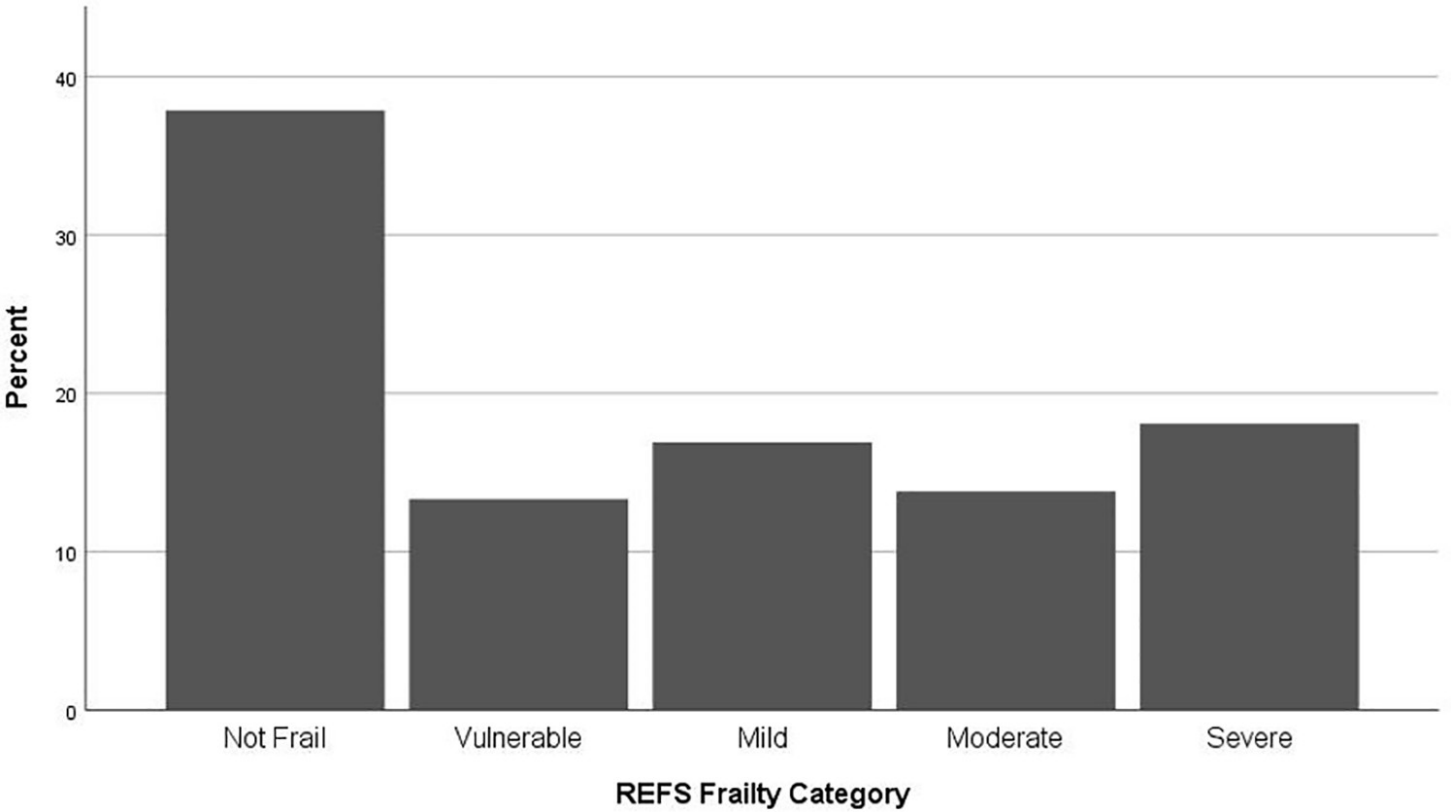
Frailty classification assessed by the Reported Edmonton Frail Scale (0–18). Patients with a score of 0–5 are classified as not frail and 6–7 as being apparently vulnerable. Patients with a score of 8–18 are classified as frail with a score of 8–9 representing mild frailty, 10–11 moderate frailty and 12–18 severe frailty.

Of the 420 patients assessed, 205 (48.8%) were classified as frail according to the REFS. (Table 1). 71 (16.9%) patients were mildly frail, 58 (13.8%) moderately frail and 76 (18.1%) were severely frail. 56 (13.3%) were apparently vulnerable. (Fig 2).

**Table 1:** Patient demographics, by frailty status.

		Total	Not Frail	Frail
		(n = 420)	(n = 215)	(n = 205)
			51.20%	48.80%
**Age**	Median	73	63	79
	IQR	58–83	49–76	68–87
	**Age Categorized**	<65	152 (36.2)	114 (53.0)	38 (18.5)
**n (%)**	65–69	33 (7.9)	17 (7.9)	16 (7.8)
	70–74	45 (10.7)	27 (12.6)	18 (8.8)
	75–79	47 (11.2)	14 (6.5)	33 (16.1)
	80–84	50 (11.9)	22 (10.2)	28 (13.5)
	≥85	93 (22.1)	21 (9.8)	72 (35.1)
**Gender**	Female	218 (51.9)	101 (47.0)	117 (57.1)
**n (%)**	Male	202 (48.1)	114 (53.0)	88 (42.9)
**Ethnicity**	NZ European	370 (88.1)	186 (86.5)	184 (89.8)
**n (%)**	Pacific	12 (2.9)	7 (3.3)	5 (2.4)
	Maori	22 (5.2)	8 (3.7)	14 (6.8)
	Asian	11 (2.6)	9 (4.2)	2 (1.0)
	Other	5 (1.2)	5 (2.3)	0 (0)
**Admission State**	Acute	385 (91.7)	194 (90.2)	191 (93.2)
**n (%)**	Elective	35 (8.3)	21 (9.8)	14 (6.8)
**Admission Service**	Medical	168 (40)	78 (36.3)	90 (43.9)
**n (%)**	Surgical	160 (38.1)	113 (52.6)	47 (22.9)
	Rehabilitation	92 (21.9)	24 (11.1)	68 (33.2)
**Baseline Level of Care**	Home	345 (82.1)	202 (94.0)	143 (69.8)
**n (%)**	Hospital	24 (5.8)	2 (0.9)	22 (10.7)
	Rest Home	51 (12.1)	11 (5.1)	40 (19.5)
**Location**	Christchurch	326 (77.6)	191 (88.8)	135 (65.9)
**n (%)**	Burwood	94 (22.4)	24 (11.2)	70 (34.1)

Frailty classified according to the reported Edmonton Frail Scale (REFS). Patients with a score of ≥8 were classified as frail.

The average age of patients included was 68.2 years (SD 19.4) and the median age 73 years (IQR 58–83). There was no significant difference between the age of those patients included compared to those excluded (p = 0.69).

Of the 420 included patients, 218 (51.9%) were female and 370 (88.1%) were New Zealand European. The majority of patients (91.7%) were admitted acutely. A total of 168 patients (40%) were admitted under a medical specialty, 160 (38.1%) under a surgical specialty and 92 (21.9%) to rehabilitation. 82.1% of patients came from their own home, 12.1% from rest home level care and the remainder from hospital level care.

**Fig 3:**
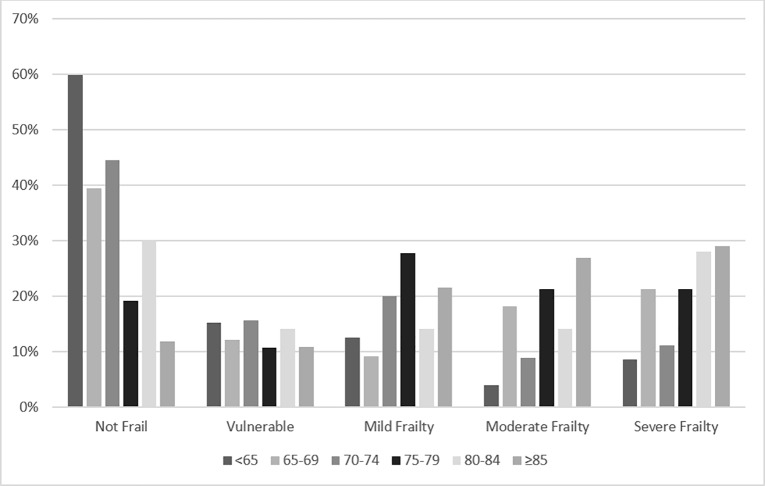
Frailty category by age category. Patients with a score of 0–5 are classified as not frail and 6–7 as being apparently vulnerable. Patients with a score of 8–18 are classified as frail with a score of 8–9 representing mild frailty, 10–11 moderate frailty and 12–18 severe frailty.

The prevalence of frailty increased significantly with increasing age (Fig 3). Of 152 patients aged under 65 years, 38 (25%) were classified as frail, compared to 72 (77.4%) of 93 patients aged 85 years or older. Frail patients were significantly older than non-frail patients with median ages of 79 and 63 years respectively (p <0.01). Patients aged 85 years and older had an odds ratio of being frail of 6.25 (95% CI 3.17–12.7) when compared to those aged under 65. (Table 2). With increasing age, patients are also more likely to have a higher degree of frailty (Fig 3).

**Table 2:** Multivariate adjusted association between demographic characteristics and frailty status.

Patient variables		Adjusted Odds Ratio^a^	p-value
		(95% CI)	
Age (per additional year)		1.04 (1.03–1.06)	<0.01
**Age Categorized**	<65	Ref	
**n (%)**	65–69	2.25 (0.94–5.34)	0.07
	70–74	1.67 (0.77–3.59)	0.19
	75–79	**5.71 (2.59–13.1)**	**<0.01**
	80–84	**2.90 (1.37–6.21)**	**0.05**
	≥85	**6.25 (3.17–12.7)**	**<0.01**
**Gender**	Female	Ref	
**n (%)**	Male	0.96 (0.61–1.52)	0.87
**Ethnicity**	NZ European	Ref	
**n (%)**	Pacific	1.70 (0.45–6.13)	0.42
	Maori	**4.00 (1.45–11.9)**	**0.01**
	Asian	0.86 (0.11–4.20)	0.87
	Other	-	-
**Admission State**	Acute	Ref	
**n (%)**	Elective	1.47 (0.63–3.40)	0.37
**Admission Service**	Medical	Ref	
**n (%)**	Rehab	**1.86 (1.03–3.41)**	**0.04**
	Surgical	**0.52 (0.31–0.86)**	**0.01**
**Baseline Level of Care**	Home	Ref	
**n (%)**	Hospital	**9.62 (2.68–61.6)**	**<0.01**
	Rest Home	**2.81 (1.38–6.14)**	**<0.01**
**Admission Location**	Christchurch	Ref	
**n (%)**	Burwood	**2.57 (1.48–2.54)**	**<0.01**

Odds Ratios comparing characteristics between frail patients (REFS ≥8) and non-frail

patients.

^a^Model adjusted for Age (as a continuous variable), Ethnicity, Admission Service, Admission

Location and Baseline level of care.

Female patients were more likely to be frail with 117 of 218 (53.9%) female patients being classified as frail compared to 88 of 202 (43.6%) male patients, (p = 0.04). After multivariate modelling, this difference was not statistically significant. The majority of patients were New Zealand European, which is representative of the local population. 49.7% (n = 184) of the New Zealand European population were frail, 41.7% (n = 5) of the Pacific population, 63.6% (n = 14) of the Maori population and 18.2% (n = 2) of the Asian population. Maori patients had a significantly higher rate of frailty when compared to the New Zealand European population (OR = 4.00, 95% CI 1.45–11.90, p = 0.02). Asian patients had a trend towards reduced levels of frailty (OR = 0.86), and Pacific patients had a trend towards increased levels of frailty (OR = 1.70), but neither were statistically significant, likely limited by the small sample size.

The prevalence of frailty differed significantly by admitting service (Tables 1 and 2). Patients admitted to a surgical service were significantly less likely to be frail, (OR = 0.52, 95% CI 0.31–0.86, p = 0.01). Those admitted to Burwood Health Campus for rehabilitation were significantly more likely to be frail. Patients admitted from care facilities, including rest homes and hospital level care, were more likely to be frail than those admitted from their own home (Table 2).

Multivariate analysis was conducted using backward stepwise selection to assess the association between demographic factors and frailty. All factors were initially included in the model and after backward stepwise selection age, ethnicity, admission service and baseline level of care remained significant predictors of frailty, with age being the strongest predictor (p <0.01). Adjusted Odds Ratios are presented in Table 2.

## Discussion

This study highlights the high prevalence of frailty within an adult inpatient population, with 48.8% of patients being classified as frail. On multivariate analysis, multiple factors were found to be predictive of frailty, with increasing age being the strongest predictor with an Odds Ratio of 1.04 per each additional year, (p <0.01).

The prevalence of frailty identified in this study is in keeping with rates of between 24.7% and 80% reported in other publications[13–18]. Prior studies[15, 17] have typically assessed the prevalence of frailty in elderly patients across a subset of inpatient services. To our knowledge, this study is the first to assess frailty across an entire adult inpatient population; and although high rates were expected and seen in the elderly cohort, we have highlighted a significant burden of frailty in younger patients. To date, although frailty is well described in the elderly patient, there is paucity of literature describing frailty in younger individuals.

Although frailty is strongly associated with increasing age, frailty is not a unique condition limited to the elderly. Smart et al[25], in a study of 82 younger adults admitted acutely to a surgical service identified 16% of their cohort to be frail. Frailty in the younger patient was associated with polypharmacy, multi-morbidity, cognitive impairment and social deprivation.

Consistent with these findings, in our cohort, 25% of patients aged under 65 were classified as frail, with the youngest frail patient being aged 25. These younger patients typically had chronic health conditions and scored highly on the general health, independence, and performance categories of the REFS.

Despite the above findings, the prevalence of frailty in this younger population remains unclear[26, 27] and further research into the prevalence and implications of frailty in this group is needed. Frailty in this group, although associated with morbidity and mortality, is likely a different clinical and biological entity to that encountered in older adults[26]. Early identification and the development of interventions to improve outcomes in this cohort is needed.

Most data in the literature has been collected on Caucasian populations and there is limited data on variations in the prevalence of frailty across ethnic groups. Fried et al[7], in the Cardiovascular Health Study, noted an increased prevalence of phenotypic frailty in the African-American cohort of 12% compared to 6% in the Caucasian cohort. Similar results were seen in the Women’s Health and Ageing Studies[28].

In our cohort, Maori patients were significantly more likely to be frail, (OR = 4.00, 95% CI 1.45–11.9, p = 0.01) and were significantly more likely to rate their general heath as fair or poor and have multiple hospital admissions over the previous twelve months. Pacific patients had a trend towards increased frailty and Asian patients towards decreased frailty; however, due to the relatively homogeneous population ethnicity this study was not powered to identify a significant difference.

Consistent with our findings, Barrett et al[29] in a survey of 2931 individuals found a higher prevalence of frailty in a Maori population compared to a New Zealand European population, with rates of 11.5% and 7.9% respectively. They also found the prevalence of frailty in Maori aged 65 to 70 to be the same as the prevalence of frailty in non-Maori aged 81 to 84, suggesting an earlier onset of frailty in the Maori population. The higher prevalence of frailty among Maori at younger ages may be linked to a shorter life expectancy. Disparities between Maori and non-Maori over the life course can be considered to have a cumulative effect that is manifest in later years as frailty. This disparity is also the likely cause of higher rates of frailty seen in the Cardiovascular Health Study and the Women’s Health and Ageing Studies[30].

Our results are in contrast to prior outpatient based studies, which estimate the prevalence of frailty to be in the region of 10%[11]. Frailty may be defined as a clinical state of increased vulnerability across multiple organ systems, resulting in poor physiologic reserve, and thus inability to respond to stressors[7–9]. This vulnerable cohort are at risk of adverse outcomes, including acute hospital admission, and therefore not unsurprisingly a large percentage of hospital inpatients are indeed frail, as seen in this study.

Multiple frailty assessment tools exist for use in the inpatient setting[19]. The lack of a consensus definition of frailty or of a standardised assessment tool, limits comparison to other literature on frailty prevalence. Other clinically validated tools exist for use in the inpatient setting, such as the Clinical Frail Scale [31] and a number of frailty indices[32] which assess for frailty using different methods. We believe the REFS has an advantage over the other tools, as it is multidimensional and easy to perform. The Clinical Frail Scale scores patients on a scale from 1 to 9 and focusses predominantly on the physical aspect of frailty and most frailty indices are composed of a large number of items, rendering them more suitable for research use. Currently the literature on frailty suffers as a whole from this lack of consensus definition[33], and of a standardised assessment tool and should be the focus of ongoing research.

A strength of this study is all adult inpatients were eligible for inclusion, thus we were able to estimate the prevalence of frailty across the entire adult inpatient population. To our knowledge, this is the first publication to do so. By including patients aged under 65, we also were able to describe the prevalence of frailty in younger individuals, where there is currently a paucity of literature.

This study has a number of limitations. Firstly, 89 patients were absent from their bed-space at the time of the study. Although we could account for some of these individuals being away at investigations and undergoing procedures, it is possible the remainder were well and ambulatory, and therefore likely less to be frail than the included population. This may have led to an overestimation of the true prevalence of frailty due to sampling error. Secondly, data were derived from a single institutional experience on a single day with a relatively homogeneous ethnicity, and this therefore may not be generalisable to other centres. Finally, although data on frailty prevalence is reported, no data on clinical outcomes is currently available from this cohort, therefore we cannot conclude that patients who were classified as frail had adverse health outcomes.

## Conclusions

Frailty is highly prevalent in the inpatient hospital setting, with a prevalence of 48.8% in the studied population. Despite typically being seen in an elderly population, it is also seen in younger patients, typically those with chronic health conditions. Due to its associated with adverse health outcomes, frailty has significant resource implications and should be the focus of ongoing research towards interventions to support this vulnerable population and attenuate the progress of frailty. Consideration should be given to inpatient frailty assessment in order to identify frailty and thus target appropriate intervention.
